# Prenatal diagnosis, associated findings and postnatal outcome in fetuses with congenitally corrected transposition of the great arteries

**DOI:** 10.1007/s00404-020-05886-8

**Published:** 2020-11-20

**Authors:** Andrea Krummholz, I. Gottschalk, A. Geipel, U. Herberg, C. Berg, U. Gembruch, A. Hellmund

**Affiliations:** 1 Department of Obstetrics and Prenatal Medicine, University of Bonn, Venusberg-Campus 1, 53127 Bonn, Germany; 2 Division of Prenatal Medicine, Department of Obstetrics and Gynecology, University of Cologne, Cologne, Germany; 3 Department of Pediatric Cardiology, University of Bonn, Bonn, Germany

**Keywords:** Corrected transposition, Atrioventricular discordance, Prenatal diagnosis, Congenital heart disease, Fetus, Fetal echocardiography

## Abstract

**Purpose:**

To analyze anatomic features and associated malformations in 37 prenatally detected cases of congenitally corrected transposition of the great arteries (ccTGA) and to evaluate the prenatal course, neonatal outcome and mid-term follow-up.

**Methods:**

Retrospective analysis of prenatal ultrasound of 37 patients with ccTGA in two tertiary centers between 1999 and 2019. All fetuses received fetal echocardiography and a detailed anomaly scan. Postnatal outcome and follow-up data were retrieved from pediatric reports.

**Results:**

Isolated ccTGA without associated cardiac anomalies was found in 13.5% (5/37), in all other fetuses additional defects such as VSD (73.0%), pulmonary obstruction (35.1%), tricuspid valve anomalies (18.9%), aortic arch anomalies (13.5%), ventricular hypoplasia (5.4%) or atrioventricular block (5.4%) were present. The rate of extracardiac malformations or chromosomal aberrations was low. There were 91.9% (34/37) live births and postnatal survival rates reached 91.2% in a mean follow-up time of 4.98 years. The prenatal diagnosis of ccTGA was confirmed postnatally in all but one documented live birth and the prenatal counselling regarding the expected treatment after birth (uni- versus biventricular repair) was reassured in the majority of cases. The postnatal intervention rate was high, 64.7% (22/34) received surgery, the intervention-free survival was 36.7%, 35.0% and 25.0% at 1 month, 1 year and 10 years, respectively.

**Conclusions:**

ccTGA is a rare heart defect often associated with additional heterogeneous cardiac anomalies that can be diagnosed prenatally. The presented study demonstrates a favorable outcome in most cases but the majority of patients require surgical treatment early in life.

## Introduction

Congenitally corrected transposition of the great arteries (ccTGA) is a rare form of congenital heart disease, mainly characterized by both atrioventricular and ventriculoarterial discordance [1, 2]. The prevalence is 0.02 per 1000 live births and it accounts for less than 1% of all congenital heart defects [1–5].

In ccTGA, the right atrium drains into a right-sided morphological left ventricle through the mitral valve and the left atrium into a left-sided morphological right ventricle through the tricuspid valve (atrioventricular discordance). The morphological left ventricle is connected to the pulmonary artery, whereas the aorta arises from the morphological right ventricle (ventriculoarterial discordance). The great vessels show a parallel course without crossing, the aorta usually located anteriorly and to the left of the pulmonary trunk. This leads to a physiologically corrected blood flow, but with the morphologic right ventricle to manage systemic circulation in postnatal life.

The majority of cases is associated with additional cardiac defects, such as a ventricular septal defect (VSD), pulmonary outflow tract obstruction, tricuspid valve (TV) anomalies, dextro-/mesocardia, aortic arch anomalies and rhythm disturbances/atrioventricular block [6–9]. In contrast, the presence of extracardiac or chromosomal anomalies is low [6, 7, 9].

Whereas the prenatal detection of major associated cardiac lesions and the abnormal origin and orientation of the great arteries is often achieved [6, 7, 10], diagnosing the double discordance might still be challenging.

Only few studies have described the prenatal finding of a ccTGA in fetuses so far [6–9, 11]. However, providing the diagnosis before birth is essential for better counselling of the parents and postnatal interdisciplinary care. While the perinatal outcome is mostly favorable [6, 8, 9, 11], in the further postnatal course development of right ventricle dysfunction, TV regurgitation, complete heart block and congestive heart failure is common [12, 13]. It leads to a high rate of surgery, but shows a variable course depending on associated anomalies [8, 10, 13].

The goal of our study was to assess the prenatal diagnosis emphasizing on echocardiographic features and associated cardiac and extracardiac malformations in a series of intrauterine diagnosed ccTGA. Furthermore, the detailed prenatal course, neonatal outcome and mid-term follow-up were evaluated.

## Subjects and methods

All cases of congenitally corrected transposition of the great arteries diagnosed prenatally in the databases in two tertiary referral centers for prenatal medicine and fetal echocardiography (Universities of Bonn and Cologne, Departments of Obstetrics and Prenatal Medicine) were retrospectively reviewed for intrauterine course and outcome between 1999 and 2019. Patients were referred for screening in high-risk population or with suspicion of fetal anomalies. In the study period, all patients received a complete fetal anatomic survey that included fetal echocardiography and Doppler sonography. The study was approved by the data protection officer and the institutional research ethics board (Ethik-Kommission der Medizinischen Fakultät, Rheinische Friedrich-Wilhelms-Universität Bonn, Bonn, Germany, No. 350/18). All data were collected during routine clinical examinations. The patients consented to the use of their data for scientific studies and publication.

**Fig. 1:**
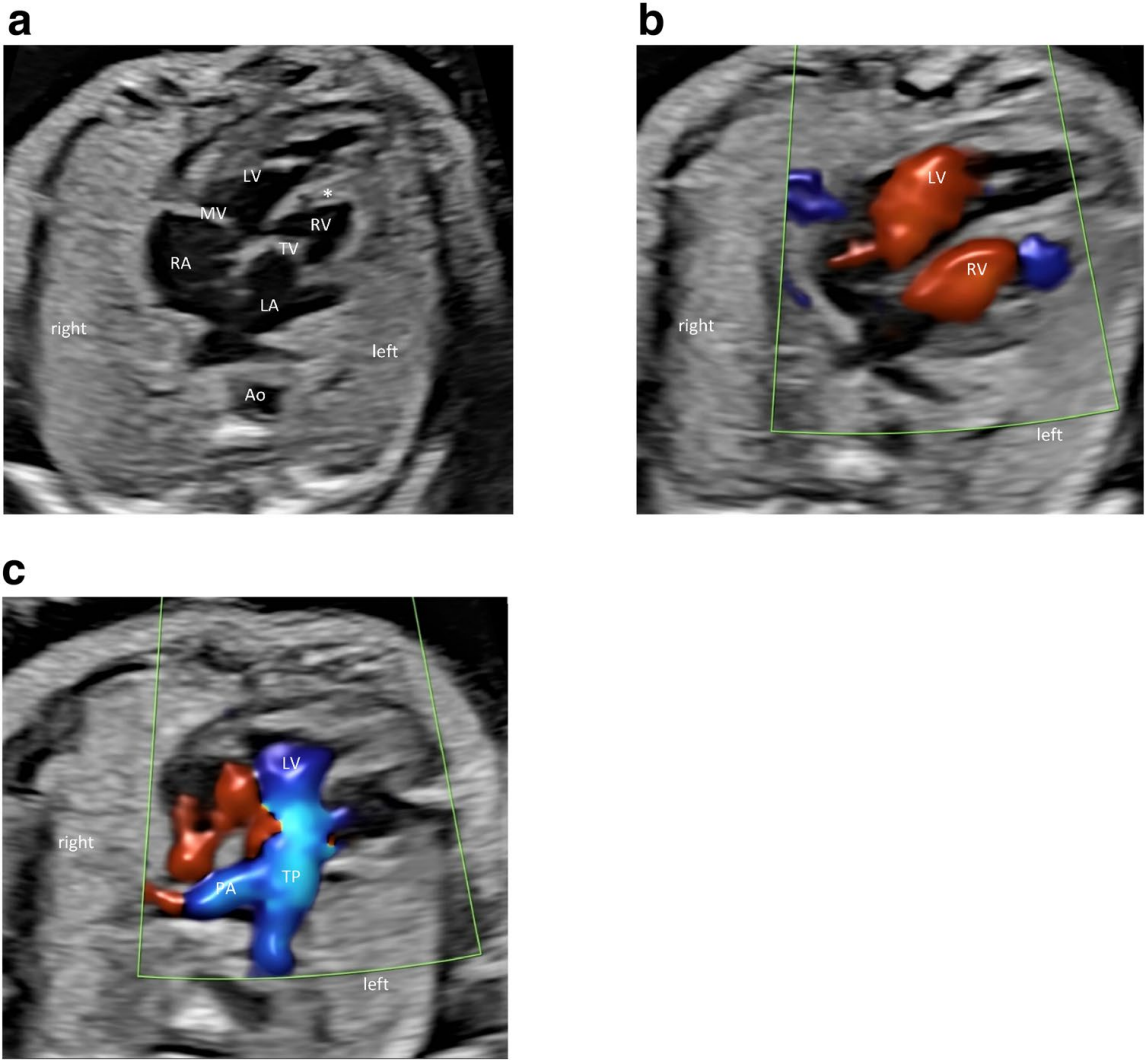
**a** Four-chamber view in a fetus with isolated ccTGA (24 + 2 weeks): levocardia, the aorta descendens (Ao) is located to the left, pulmonary veins drain into the left atrium (LA), connected to the left-sided anatomic right ventricle (RV), where the moderator band (asterisk) is found and the septal leaflet of the tricuspid valve (TV) inserts lower to the interventricular septum than the anterior leaflet of the mitral valve (MV); the right atrium (RA) is connected to the right-sided anatomic left ventricle (LV), that forms the apex of the heart. **b** Diastolic inflow in left (LV) and right (RV) ventricle. **c** From the right-sided left ventricle (LV) arises the pulmonary trunk (TP), the blood flow separates into the pulmonary arteries (PA) and the ductus arteriosus

The diagnosis of ccTGA was defined as both an atrioventricular and ventriculoarterial discordance and a malposition and parallel course of the great vessels in prenatal echocardiography. Therefore, fetal echocardiography was conducted in a standardized way with a segmental approach using standardized anatomical planes in combination with color and pulsed-wave Doppler imaging [14–16] (see also Fig. 1). All examinations were performed using 5-MHz, 7.5-MHz or 9-MHz sector or curved-array probes (ATL HDI 5000 and IU22 and EPIQ7 Philips, Hamburg, Germany; Aplio i900 Toshiba, Minato, Japan; Voluson E8, E6, S6, E10, 730 expert GE Healthcare, Solingen, Germany; Sonoace V20 Samsung, Suwon, South Korea; Acuson Aspen Siemens, Munich, Germany). Special attention was paid to atrioventricular and ventriculoarterial connection/concordance, position of the great vessels and outflow tract obstruction as well as associated further cardiac anomalies. At both the centers, prenatal ultrasound examinations were followed by counselling of the parents by a pediatric cardiologist. Follow-up scans were performed at 2–6-week intervals. The fetuses were delivered in tertiary care centers. All neonates received initial care by a neonatologist followed by an examination by a pediatric cardiologist within 12 h.

Data were obtained from reviewing medical files, stored ultrasound images and—if available—ultrasound video recordings. Regarding pregnancy, outcome and postnatal course pediatric (neonatology and cardiology) reports and medical files as well as surgical protocols were analyzed. Except for three cases—one lost to follow-up, two terminations of pregnancy (TOP)—, follow-up was available between 1 day and 18 years after birth. It was evaluated for Apgar score, birth weight, confirmation of prenatal diagnosis, additional postnatal diagnoses, performance of surgery, death in neonatal period, infancy or childhood. The prenatal diagnosis of ccTGA was confirmed postnatally in all but one documented live birth by echocardiography and, if performed, cardiac surgery. There was no autopsy conducted in the two cases of TOP due to refusal of the parents.

Data analyses were performed using the Statistical Package for Social Sciences (SPSS 22.0 INC. Chicago, III USA), but limited to descriptive analysis.

## Results

### Patient population

Between January 1999 and December 2019, more than 145,000 pregnancies were examined by ultrasound in the two tertiary centers. In this period, 37 cases of ccTGA were diagnosed prenatally. Detailed information is presented in Table 1.

**Table 1:** Patient population

Total cases	37
Lost to follow-up	1 (2.7%)
Termination of pregnancy	2 (5.4%)
Live birth	34 (91.9%)
Mean follow-up time	4.98 years = 59.7 months (1 day–18 years)
Overall survival rate	31/37 (83.8%)
Adjusted survival rate (without TOP and lost to follow-up)	31/34 (91.2%)
Neonatal death	2/34 (5.9%)
Death in infancy	1/34 (2.9%)
Isolated ccTGA	5/37 (13.5%)
Associated anomalies	32/37 (86.5%)
Mean maternal age	30.4 years (19–44)
Mean gestational age at diagnosis	22 + 4 weeks (11 + 3–35 + 2)
Mean gestational age at delivery	38 + 4 weeks (33 + 4–41 + 2)
Fetal sex	15 females (40.5%) 22 males (59.5%)
Mean birth weight	3179 g (1390–3840 g)

Reasons for referral to the tertiary centers were the suspicion of a cardiac defect in the majority of cases (78.4%, 29/37), furthermore screening in a high-risk population (13.5%, 5/37), fetal hydrops in a previous pregnancy (2.7%, 1/37), diagnosis of extracardiac fetal anomalies (2.7%, 1/37) and an enlarged nuchal translucency (2.7%, 1/37).

Karyotyping was performed in 45.9% (17/37) and in only 1 case an abnormal result occurred (trisomy 13). No other abnormalities, such as microdeletion 22q11, were found. Although less than half of the patients received karyotyping, none of the neonates showed external features that suggested chromosomal aberrations or received karyotyping postnatally.

### Prenatal ultrasound findings

An isolated form of a discordant atrioventricular and ventriculoarterial connection without additional structural cardiac malformations was seen in eight cases (21.6%, 8/37). Of those, two showed dextrocardia and two mesocardia. There was a situs solitus in the majority of cases (83.8%, 31/37). However, five fetuses showed a situs inversus visceralis with levocardia (13.5%, 5/37) and one a situs ambiguus (2.7%, 1/37).

The majority of fetuses presented associated structural cardiac malformations (78.4%, 29/37), as listed in Table 2.

**Table 2:** Associated cardiac anomalies

Associated anomaly	%	%
	Prenatal cohort (*n* = 37)	Postnatal cohort (*n* = 34)
Ventricular septal defect	73.0 (*n* = 27)	73.5 (*n* = 25)
Pulmonary obstruction	35.1 (*n* = 13)	55.9 (*n* = 19)
Pulmonary atresia (PA)	*n* = 7	*n* = 7
Valvular stenosis	*n* = 2	*n* = 5
Infravalvular stenosis	*n* = 4	*n* = 7
Tricuspid valve defect	18.9 (*n* = 7)	23.5 (*n* = 8)
Tricuspid atresia	*n* = 1	*n* = 1
Ebstein anomaly	*n* = 3	*n* = 2
Dysplastic TV	*n* = 1	*n* = 2
Straddling of TV	*n* = 2	*n* = 3
Aortic arch anomalies	13.5 (*n* = 5)	29.4 (*n* = 10)
Hypoplasia/coarctation	*n* = 1	*n* = 3
Right aortic arch	*n* = 3	*n* = 6
Double aortic arch	*n* = 1	*n* = 1
Hypoplastic right ventricle	5.4 (*n* = 2)	2.9 (*n* = 1)
Left-persistent superior vena cava (LPSCV)	5.4 (*n* = 2)	8.8 (*n* = 3)
Dextro-/mesocardia	29.7 (*n* = 11)	29.4 (*n* = 10)
Dextrocardia	*n* = 7	*n* = 7
Mesocardia	*n* = 4	*n* = 3
Atrioventricular block	5.4 (*n* = 2)	11.8 (*n* = 4)
Tricuspid regurgitation (without structural defect)	2.7 (*n* = 1)	11.8 (*n* = 4)

The atrioventricular block (AVB) found in 2 fetuses (5.4%, 2/37) was present at the first examination in 25 + 5 weeks (AVB II°) and 29 + 6 weeks (AVB III°), respectively.

Ultrasound examinations prior to birth were performed at 4–6-week intervals, in some cases up to 2 weeks. There was no progression within the prenatal course and no intrauterine decompensation. In four cases, the echocardiographic diagnosis was changed at the repeat scan: in two cases, a double-outlet right ventricle (DORV) and in one case, a suspicion of tetralogy of Fallot (TOF) were described in early pregnancy and corrected to ccTGA at 20–25 weeks; in one case the diagnosis “suspicion of DORV or TOF” was changed to ccTGA prenatally at 24 weeks but a malposition of the great arteries in combination with DORV was diagnosed postnatally.

Extracardiac anomalies were found in 13.5% (5/37) prenatally. Multiple malformations were detected in the fetus with trisomy 13 (Dandy–Walker malformation, hexadactyly, enlarged hyperechogenic kidneys, micrognathus, growth restriction, and rocker-bottom feet). Other findings in solitary cases were: agenesis of the ductus venosus, persistence of right umbilical vein, azygos vein continuity and unilateral pes equinovarus.

### Outcome

There were 34 live births (91.9%, 34/37). TOP was performed in two cases (5.4%, 2/37): one in 28 + 2 the abovementioned pregnancy with fetal trisomy 13; one in 23 + 0 with Ebstein anomaly (Carpentier C) with a hypoplastic right ventricle and severe tricuspid regurgitation (TR). An autopsy was not performed in both the cases. No intrauterine death or death during birth occurred.

One case (2.7%, 1/37) is lost to follow-up with missing information after the last prenatal ultrasound in 35 + 6 weeks.

Mode of delivery was a cesarean section in 44.1% (15/34) and a vaginal birth in 55.9% (19/34). Although the rate of cesarean delivery (CD) seems high, a primary CD was only performed in 7/15 cases, in the remaining 8 cases it was a secondary CD due to subpartal complications. The reasons for a primary CD were IUGR, HELLP, preeclampsia, state after cesarean or breech position. ccTGA alone was no reason to give the advice for a CD, whether it was a contributing factor cannot be deduced with certainty from the existing documentation.

Out of 34 live born, 31 survivors were reported, 2 neonatal deaths (NND; 5.9%, 2/34) within the first 28 days after birth and 1 death in infancy (2.9%, 1/34) 13 months after birth. The latter case presented with associated VSD, pulmonary stenosis (PS), severe TR and complete heart block (CHB) after cardiac catheter intervention; Glenn procedure was performed at the age of 6 months and pacemaker (PM) implantation at 7 months but death occurred at 13 months due to cardiac failure. The two NND both occurred 28 days after birth and several days after surgical procedures. The first newborn with VSD and dysplastic TV with mild TR developed a supraventricular tachycardia and received an arterial switch operation at the age of 15 days, completion of double switch was planned 6 months later. However, postoperative complications led to death due to cardiogenic shock. The second newborn with postnatally detected aortic coarctation (prenatally isolated case of ccTGA) underwent reconstruction of the aortic arch 27 days after birth and died due to acute cardiac failure 1 day later.

The prenatal diagnosis of ccTGA was confirmed postnatally in 33 cases (89.2%, 33/37). No confirmation was achieved in four cases (one lost to follow-up, two TOP without autopsy, and one the abovementioned case of malposition of the great arteries in combination with DORV). However, there were additional findings postnatally regarding associated structural cardiac anomalies in 12 cases (35.3%, 12/34) (Table 3).

**Table 3:** Associated anomalies and outcome

Cases	Dextroposition	VSD	Pulmonary obstruction	Tricuspid valve anomalies	Rhythm disturbances	Others	Extracardiac anomalies	Additional cardiac anomalies postnatally	Mode of delivery, gestational age at birth in weeks	Live birth, Apgar score 5–10 min	Outcome
1		×	Infravalvular stenosis	Ebstein					Vaginal, 38 + 1	live birth 10–10	Alive at 3 weeks, no therapy
2		×		Straddling				Mild CoA (at 1 month)	Cesarean, 37 + 0	Live birth 7–10	Alive at 2 years, PA banding, Glenn
3		×		Atresia					Cesarean, 39 + 0	Live birth 9–10	Alive at 13.5 years, a-p shunt, Glenn, TCPC, atrial PM
4									Cesarean 38 + 4	Live birth 10–10	Alive at 3 years, captopril
5		×	Pulmonary atresia				Situs inversus, agenesis of ductus venosus		Cesarean, 37 + 1	Live birth 9–9	Alive at 6 months, a-p shunt, Glenn planned, West syndrome
6		×		Ebstein with severe TR		Hypoplastic RV			TOP, 23 + 0	TOP	
7	Mesocardia						Multiple, trisomy 13		TOP, 28 + 2	TOP	
8			Valvular stenosis						Vaginal, 37 + 3	Live birth 8–9	Alive at 5 years, lisinopril
9		×						Infravalvular PS, moderate TR	Vaginal, 39 + 4	Live birth 10–10	Alive at 14.5 years, enalapril
10									Vaginal, 40 + 0	Live birth 10–10	Alive at 6 months, lisinopril
11	Dextrocardia							Mild valvular PS	Vaginal, 37 + 5	Live birth 10–10	Alive at 7 years, ASD occlusion
12		×						Infravalvular PS, AVB III° at 6 months, TV straddling + severe TR	Vaginal, 40 + 3	Live birth 9–9	PM, Glenn, death in infancy at 13 months
13		×		Dysplastic valve		Hypoplastic RV			Vaginal, 39 + 4	Live birth 10–10	Alive at 10 years, PA banding, Glenn, TCPC
14	Mesocardia		Infravalvular stenosis				Situs inversus		Cesarean, 33 + 4	Live birth 10–10	Alive at 2 months, no therapy
15		×				Double aortic arch	Situs inversus	Valvular PS	Vaginal, 38 + 0	Live birth 9–10	Alive at 5 years, valvuloplasty PV
16		×							Cesarean, 34 + 5	Live birth 8–9	Alive at 11 years, repair of associated lesion
17	Mesocardia	×	Infravalvular stenosis					Right aortic arch	Cesarean, 36 + 4	Live birth 9–10	Alive at 18 years, repair of associated lesion
18	Dextrocardia	×	Pulmonary atresia					LPSVC	Cesarean, 39 + 0	Live birth 10–10	Alive at 5.5 years, a-p shunt, Senning-Rastelli
19		×				LPSCV, right aortic arch	Situs inversus	Infravalvular PS	Vaginal, 41 + 2	Live birth 10–10	Alive at 6 days
20	Dextrocardia						PRUV	Right aortic arch	Vaginal, 39 + 3	live birth 7–9	Alive at 11 years, no therapy
21		×				Hypoplastic aorta	Situs ambiguus, azygos vein continuity		Vaginal, 38 + 2	Live birth 8–8	Alive at 14.75 years, Norwood-Sano, Kawashima, TCPC
22	Dextrocardia	×	Pulmonary atresia						Vaginal, 38 + 4	Live birth 9–9	Alive at 6 years, a-p shunt, VSD occlusion and LVPA conduit
23		×	Pulmonary atresia			Right aortic arch	Situs inversus		Cesarean, 38 + 1	Live birth 9–10	Alive at 4 years, a-p shunt, Glenn, TCPC
24		×			AVB II°			Moderate TR	Cesarean, 37 + 6	Live birth 9–10	Alive at 2 months, captopril
25		×	Pulmonary atresia	Moderate TR					Vaginal, 40 + 2	Live birth 9–10	Alive at 9 years, a-p shunt, Glenn, TCPC
26	Dextrocardia	×	Pulmonary atresia			LPSCV			Cesarean, 37 + 2	Live birth 9–10	Alive at 1 month, a-p shunt
27	Dextrocardia	×	Pulmonary atresia					Postoperative AVB III° at 10 months	Vaginal, 40 + 2	Live birth 10–10	Alive at 5.75 years, a-p shunt, LVPA conduit, PM
28		×	Infravalvular stenosis			Right aortic arch		DORV	Cesarean, 40 + 2	Live birth 9–9	alive at 17 years, TCPC
29		×		Straddling					Vaginal, 40 + 5	Live birth 10–10	Alive at 4 months, Norwood-Sano
30		×		Ebstein with moderate TR					Vaginal, 39 + 2	Live birth 10–10	Alive at 1 year, PA banding, double switch planned
31	Dextrocardia	×						Valvular PS	Vaginal, 39 + 3	Live birth 9–9	Alive at 1 day
32								CoA	Cesarean, 39 + 2	Live birth 10–10	NND 28 days
33		×						Supraventricular tachycardia, right aortic arch, dysplastic TV	Vaginal, 37 + 2	Live birth 9–9	NND 28 days
34									Vaginal, 40 + 6	Live birth 10–10	Alive at 14 months, no therapy
35		×			AVB III			Unknown	Unknown	Unknown	Unknown
36		×	Valvular stenosis					Spontaneous AVB III° at 1 month	Cesarean, 38 + 6	Live birth 9–9	Alive at 3 months, a-p shunt and PM
37	Mesocardia						Pes equinovarus		Cesarean, 37 + 5	Live birth 6–8	Alive at 4 days

*VSD* ventricular septal defect, *TR* tricuspid regurgitation, *RV* right ventricle, *PS* pulmonary stenosis, *TOP* termination of pregnancy, *TV* tricuspid valve, *AVB* atrioventricular block, *LPSCV* left-persistent superior caval vein, *CoA* aortic coarctation, *DORV* double-outlet right ventricle, *SVES* supraventricular extrasystole, *PA* pulmonary artery, *a-p* aorto-pulmonary, *TCPC* total cavo-pulmonary connection, *PM* pacemaker, *ASD* atrial septal defect, *PV* pulmonary valve, *LVPA* left ventricle-to-pulmonary artery, *NND* neonatal death

There were five cases of AVB (Table 3): the two abovementioned prenatal cases, AVB II (persisted at the same degree after birth) and AVB III° (lost to follow-up); one spontaneous postnatal AVB (manifestation at 1 month), one after diagnostic cardiac catheter intervention at 6 months and one postoperative complete AVB at the age of 10 months. In addition, another neonate developed a supraventricular tachycardia postnatally at the age of 7 days.

Thus, in combination of pre- and postnatal findings, there were only five isolated cases of ccTGA without additional intracardiac malformations (13.5%, 5/37).

Follow-up was available between 1 day and 18 years after birth, the mean follow-up time was 4.98 years. The majority of patients underwent surgery (64.7% of live birth, 22/34). Of the 12 cases without any operation, 5 received long-term medication with ACE inhibitor (follow-up range 2 months to 13 years) and 7 did not obtain any therapy (follow-up range 1 day to 11 years). However, in cases of short follow-up, therapeutic interventions or medication in the further course of life could not be described.

36.7% of the neonates had not received any intervention at the age of 1 month, 35.0% at 1 year and 25.0% at 10 years of age.

In the prenatal counselling by the pediatric cardiologist in 27 cases, it was documented, whether an univentricular or biventricular situation was present and to be expected after birth and what kind of surgical treatment would be suitable/necessary postnatally. Reasons for a univentricular approach, assessed in eight cases, were straddling (two) or atresia (one) of TV, large VSD (three) or hypoplastic ventricle (one). In 19 cases, a biventricular repair—if necessary—was assessed.

**Fig. 2:**
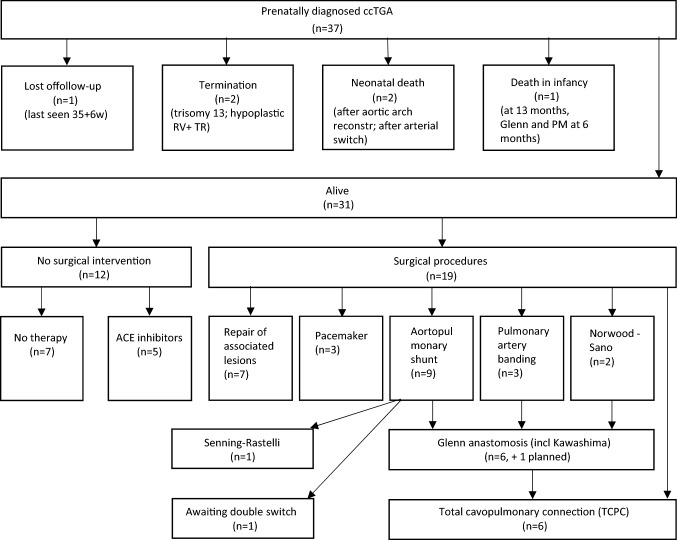
Outcome

Surgical procedures performed were minimal invasive interventions by catheter only in two cases (ASD occlusion; valvuloplasty of pulmonary valve) as well as complex cardiac operations, as shown in Fig. 2. Among the live births, ten univentricular repairs were performed, partially or completed (29.4%, 10/34), of which seven received a correct prenatal counselling (70.0%, 7/10). In the remaining three cases, prenatally a biventricular repair was favored but changed postnatally due to small RV (two) or straddling of TV detected after birth (one). Three infants required pacemaker implantation within the first year, one an atrial pacemaker at the age of 10 years (due to symptomatic bradycardia). Other surgical procedures were: aortic arch reconstruction, arterial switch (in preparation of double switch), VSD and/or ASD occlusion, LVPA conduit, transection of the (non-patent) ductus arteriosus leading to a tracheal stenosis (Fig. 2).

## Discussion

Due to the rarity of ccTGA, we still know little about the spectrum and the outcome of prenatally diagnosed cases. There are case reports and few retrospective series with a small number of patients similar to our study, one larger study of 98 cases published in 2019 [8] and a series of 69 cases in 2020 [9]. Sharland et al. reported on a series of 34 cases focusing on prenatal diagnosis. However, the study contains little data concerning the time of follow-up or therapeutical interventions [7]. The study of Paladini et al. included 30 prenatally detected cases with a short-term follow-up (median 32 months) [6]. Wan et al. reported on 16 prenatal cases, whereas the majority of cases (39) was diagnosed postnatally [11]. Day et al. presented the largest cohort of prenatally diagnosed patients with discordant atrioventricular and ventriculoarterial connections including a postnatal median follow-up of 9.5 years [8]. In their multicenter report, Vorisek et al. included 69 fetuses with ccTGA, focusing on the prenatal sonographic features and the confirmation postnatally while there were few information regarding the outcome and follow-up time was short (30 days) [9]. Our study comprises data from 37 prenatally diagnosed fetuses with detailed information regarding time of diagnosis, intrauterine course, maternal characteristics, additional cardiac and extracardiac malformations and a mid-term follow-up (Table 4).

**Table 4:** Literature review

	Period of data collection	No. of cases	Week of pregnancy at diagnosis, mean (range)	No associated cardiac defects (“isolated” ccTGA)	Live birth	Cases with postnatal follow-up	Time of follow-up, mean (range)	Survival	Rate of intervention
Sharland et al. [7]^a^	1993–2003	34	20 (15–31)	14.7%	23 (67.6%)	23	Not enumerated	82.6% of live birth	Not enumerated
Paladini et al. [6]	1994–2003	30	25.5 (21–38)	13.3%	24 (80%)	24	32 months	87.5% of live birth	45.8% (11/24)
Wan et al. [11] (prenatal cohort)	1999–2006	16	20 (16–37)	Not enumerated	14 (87.5%)	14	23 months (0.7–68 months)	86% at 5 years	64% (9/14)
Day et al. [8]^a^	1989–2018	98	21 (14–36)	15.3%	51 (52%)	43	9.5 years (36 days–22.7 years)	80% at 5 years	53.5%
Vorisek et al. [9]	2002–2017	69	25.6 (20–32)	13.0%	58 (84.1%)	58 (52 ccTGA)	30 days	94.2% at 30 days	44%
This series	1999–2019	37	23 (12–36)	13.5% (21.6% prenatally)	34 (91.9%)	34	4.98 years (1 day–18 years)	91.2% of live birth	64.7%

^a^Annotation: The cases described in the publication of Sharland et al. are also included in the publication of Day et al.

Diagnosing ccTGA in prenatal ultrasound is possible with a good accuracy, especially in specialized centers, although it remains challenging, in particular when associated defects are missing [6, 10]. Focusing on the differentiation of the left and right ventricles in echocardiography is recommended, e.g., identifying the morphologic right ventricle in the four-chamber view by a posterior and left position, a prominent moderator band, a more irregular endocardial surface, more apical attachment of the atrioventricular (tricuspid) valve and distal and central attachment of the papillary muscles. In contrast the morphological left ventricle is characterized by a smooth surface, a more elongated shape, a less apical inserted mitral valve and papillary muscles that attach to the side wall of the ventricle [6, 7, 10]. This identification is important, particularly when a parallel course of the great arteries is found, to distinguish ccTGA from complete transposition of the great arteries (d-TGA), a cardiac anomaly that requires a different management directly after birth.

Prenatal cardiac findings in our cohort are in fair agreement with previous studies. Isolated ccTGA without associated cardiac defects formed a minority with 21.6% prenatally, described before 13.0–15.3% [6–9]. Taking additional postnatal findings into account, the rate was even lower (13.5%). Most cases showed one or more structural cardiac anomaly, resulting in a heterogeneous and often complex heart disease.

A ventricular septal defect was the most common associated lesion, followed by an obstruction of the pulmonary outflow tract. In seven fetuses, pulmonary atresia and in six fetuses, pulmonary stenosis was correctly diagnosed prenatally; furthermore, there were six cases of mild pulmonary stenosis seen postnatally that were not detected in utero. This can be explained by the different circulation in fetus and neonate with the increased pulmonary blood flow after birth.

Another anomaly affects only few fetuses with ccTGA, but has an influence on the outcome of those are rhythm disturbances, especially atrioventricular block. This can occur in utero as well as postnatally, spontaneously or postoperative; an AVB II° might develop to AVB III° (CHB) over time. AVB is described to worsen the outcome of fetuses with ccTGA. A not significant trend towards a worse outcome in patients with AVB prenatally was reported [8] and in postnatal cohorts, it is one of the risk factors for developing congestive heart failure in long-term course [12, 13]. All cases developed after birth in our series required pacemaker implantation. In previous prenatal series, a similar incidence is described [6–8, 11], a higher rate was found in the study of Vorisek et al. (18.5%) [9].

Repeatedly diagnosed are abnormalities of the tricuspid valve in 20–35% of prenatal series [6–8, 11], mostly Ebstein anomaly or Ebstein-like attachment (lower than normal), often in combination with a valve insufficiency leading to regurgitation (TR), but also TV dysplasia or TV atresia. Day et al. mention antenatal TR as a risk factor for higher mortality even in mid-term postnatal follow-up with two deaths out of three live births within the first 4 months [8]. In our series, we found a similar small number of TR (Table 2). While the case with prenatally severe TR resulted in TOP, the other patients did not show a poorer outcome after birth (at a follow-up time of 3 weeks, 1 year and 9 years). The course was similar in the three newborns developing TR postnatally: one patient with severe TR died (at 13 months of age), while two patients with moderate TR had a good outcome without surgery at the age of 13 years and 2 months, respectively. Although the number of patients is small, these findings underline the fact that severe TR increases the risk of surgical intervention and death not only in long-term course but already within in first year of postnatal life, whereas mild to moderate TR not necessarily impairs the short- and mid-term outcomes.

Another feature worth attending to is dextro-/mesocardia. It was found in 29.7% in our series, very similar to 27.8% in Vorisek et al. [9]. Paladini et al. reported 16.6% prenatally [6], while in postnatal cohorts a rate up to 30% [12] is described. Therefore, our data confirm that dextrocardia is a common finding in ccTGA patients and might be a hint for prenatal diagnosis. ccTGA should be one of the possible diagnoses when dextrocardia is detected. In addition, a situs inversus visceralis is not an uncommon finding [17]. We found a rate of 13.5% with no affection on the outcome.

As described before [6, 7, 9], the rates of chromosomal aberrations as well as extracardiac malformations are low. In our study, only one case of trisomy 13 was diagnosed, representing the single case in both chromosomal and multiple extracardiac anomalies. Although only 45.9% of the patients received karyotyping, neither in neonatal period nor later in postnatal life any suspicion of chromosomal defects occurred. Apart from the fetus with trisomy 13, the extracardiac anomalies detected were seen in fetuses with normal karyotype. In consequence, karyotyping in prenatally diagnosed ccTGA without other noticeable findings does not seem obligatory. However, testing for microdeletion 22q11 might be recommended [17] and further investigation by CGH array should be discussed with the parents.

No aggravation or even decompensation in utero was seen; that was also described in previous studies [6, 11]. The rate of TOP was low (5.4%) compared to other studies (39.8% [8], 32.0% [7], and 16.6% [6]), although the spectrum of associated cardiac and extracardiac anomalies as well as the gestational age at diagnosis seem to be similar. There was no need to cause a premature birth due to ccTGA. 3/35 deliveries were prior to 37 + 0 weeks (preeclampsia, preeclampsia with HELLP syndrome and premature labor, respectively), only one of those before 34 + 0, and all newborns presented a good outcome. Therefore, prematurity did not play a significant role in our collective.

The survival after birth was 91.2% and there was no death after 13 months among the cases with an appropriate follow-up time. Surgical procedures were performed in 64.7% in our study. Basically, the surgical approach in ccTGA patients can either result in a biventricular heart, whether by anatomic repair, where the AV and VA discordance is corrected with the LV resulting in the systemic circuit, or by so-called physiologic (or conventional) repair, where the RV remains the systemic ventricle and only associated defects are corrected. Or the result is a univentricular heart, in patients with non-septatable hearts (e.g., functional single ventricle due to large VSD or ventricular hypoplasia) or in selected other cases [18]. Postnatal surgical correction resulting in a functional univentricular heart was recommended in the prenatal counselling in 7/10 cases, so the prediction was accurate in 70.0%. In the patients that were evaluated for a biventricular situation prenatally but received univentricular repair, it was for a small RV or postnatally detected TV straddling with TR. The rate of univentricular repair seems high, as well as the rate of physiologic repair, whereas in contrast, very few anatomic repairs were reported (one Senning–Rastelli procedure, one incomplete and one planned double-switch procedure). One reason might be the progressive realization of operations like the double-switch procedure with increasing data also for ccTGA patients within the past 2 decades, so that in patients diagnosed many years ago a physiologic or univentricular surgical approach was favored even in potentially septatable hearts [18, 19]. This might change with increasing follow-up time in patients not (yet) operated. Another factor may be a high rate of functional univentricular hearts in our cohort due to large VSD, anomalies of TV or hypoplastic ventricle, although the rates are comparable to the other prenatal series. To our knowledge, the prenatal recommendation of surgery was not specified in previous studies. It still appears to remain challenging in some cases showing the complexity of ccTGA patients. If any kind of operation took place, in all but three cases the first procedure was performed within the first month, often representing only one of several operations within the follow-up period, planned as well as due to complications. This results in an intervention-free survival of 36.7%, 35.0% and 25.0% at the age of 1 month, 1 year and 10 years of age, respectively. This high rate of early and subsequent intervention is also an important information for parents in prenatal counselling. In all but one prenatal series, an intervention rate higher than 40% was mentioned [6, 8, 9, 11] (Table 4).

Apart from associated cardiac defects, the main problem in ccTGA in long-term course is the gradual dysfunction of the RV in the high-pressure systemic circulation that lead to congestive heart failure (CHF) in a high percentage in adult live [12, 13, 18, 19]. Rutledge et al. found a survival rate of 92%, 91% and 75% after 5, 10 and 20 years, respectively; 71.1% (86/121) of the patients received surgery [12]. Graham et al. described CHF as common in adult patients, especially in the fourth and fifth decade, the rate increasing with age, associated lesions, arrhythmia, prior surgery and strongly associated with TR and RV (systemic ventricle) dysfunction [13]. Regarding the type of surgery, the double-switch procedure is endorsed in several studies with good survival and low complications [20, 21], especially good early and intermediate results [19]. Another study showed no statistical difference in long-term survival between conventional surgical repair and anatomical surgical repair [22]. Choosing the right patient for a double-switch procedure and the timing for the operation is mentioned to be a difficult challenge [23]. In addition, the procedure of anatomic correction is described to be a challenging complex surgery providing excellent functional outcomes in survivors, but with a significant rate of reoperation and a definite risk of death or transplantation as well as a relevant rate of postoperative AVB. [24].

No information about the mental and physical development or quality of life of the patients could be gained from previous studies. In addition, in our series, this information is scarce because the pediatric reports focus on the cardiac function. When no operation was performed or planned at the age of 1 year and older, normal physical and mental development and well-being was described. In the group of patients who received surgical intervention, the course is variable. In three patients, substandard intelligence and learning disability are documented, whether there is a connection to the cardiac defect or operations is unclear; two cases of hypoxic encephalopathy after several surgeries and complications occurred. More information needs to be obtained in future studies.

Study limitations comprise the small number of patients and the often short follow-up time in a retrospective study. Therefore, no information on long-term course can be given with this study. Further investigations on mortality, morbidity and quality of life are preferable to improve prenatal counselling.

In conclusion, ccTGA is a cardiac anomaly that can be diagnosed prenatally with a good accuracy and shows an uneventful intrauterine course, an acceptable postnatal outcome with treatment options and rarely extracardiac defects but presents a wide spectrum of associated cardiac lesions forming a heterogeneous heart disease. This fact complicates the assessment regarding the expected (surgical) treatment, but correct information can be given in the majority of cases.

## Data Availability

The datasets generated and/or analyzed during the current study are available from the corresponding author on request.
